# Lyn, a Src family kinase, regulates activation of epidermal growth factor receptors in lung adenocarcinoma cells

**DOI:** 10.1186/1476-4598-12-76

**Published:** 2013-07-16

**Authors:** Parnetta Sutton, Jeffrey A Borgia, Philip Bonomi, Janet MD Plate

**Affiliations:** 1 Department of Medical Laboratory Sciences, Rush University Medical Center, Chicago, IL 60612, USA; 2 Division of Oncology, Department of Medicine, Hematology and Cell Therapy, Rush University Medical Center, Chicago, IL 60612, USA; 3 Department of Biochemistry, Rush University Medical Center, Chicago, IL 60612, USA; 4 Department of Immunology & Microbiology, Rush University Medical Center, Chicago, IL 60612, USA

**Keywords:** Lung adenocarcinoma cells, Epidermal growth factor receptors, Src family kinases, Lyn, Membrane-associated protein complexes/lipid rafts, RACK1, Cbp\PAG

## Abstract

**Background:**

Activation of receptors for growth factors on lung epithelial cells is essential for transformation into tumor cells, supporting their viability and proliferation. In most lung cancer patients, EGFR is constitutively activated without evidence of mutation. Defining mechanisms for constitutive activation of EGFR could elucidate additional targets for therapy of lung cancers.

**Methods:**

The approach was to identify lung cancer cell lines with constitutively activated EGFR and use systematic selection of inhibitors to evaluate their effects on specific EGFR phosphorylations and downstream signaling pathways. Interactions between receptors, kinases, and scaffolding proteins were investigated by co-immunoprecipitation plus Western blotting.

**Results:**

The results revealed a dependence on Src family of tyrosine kinases for downstream signaling and cell growth. Lyn, a Src family kinase functional in normal and malignant B-lymphocytes, was a defining signal transducer required for EGFR signaling in Calu3 cell line. Src family kinase activation in turn, was dependent on PKCßII. Lyn and PKC exist in membrane complexes of RACK1 and in association with EGFR which pairs with other receptor partners. Silencing of Lyn expression with interfering siRNA decreased EGFR activation and cell viability.

**Conclusions:**

The importance of Src family kinases and PKCßII in the initiation of the EGFR signaling pathway in lung tumor cells was demonstrated. We conclude that phosphorylation of EGFR is mediated through PKCßII regulation of Lyn activation, and occurs in association with RACK1 and Cbp/PAG proteins. We suggest that protein complexes in cell membranes, including lipid rafts, may serve as novel targets for combination therapies with EGFR and Src Family Kinase inhibitors in lung cancer.

## Introduction

The ErbB epidermal growth factor family of receptors (EGFR) is often upregulated, amplified, mutated, or overexpressed in cancer cells [1-3]. EGFR is a homodimer of ErbB1, but different family members can heterodimerize with ErbB1 to yield functional partners, some more active than EGFR itself (reviewed in [2,4]), [5]. Immunohistochemical staining of normal human bronchial epithelium detects ErbB1, ErbB2 (HER2/Neu), and ErbB3 (HER3) [6]. The signaling pathways triggered by EGFR are critical to lung cancer as blocking with specific inhibitors results in cell death [7-12]. ErbB1 chains contain intracellular tyrosines some of which become autophosphorylated by dimerization and serve as docking sites for adaptor proteins that convey signals downstream thus promoting cell survival, angiogenesis, migration and tumor cell invasion [13,14]. Additional phosphorylations of EGFR by other kinases stabilize and enhance receptor activity [4,15]. The importance of EGFR kinase activity in lung cancer is illustrated by the approval of tyrosine kinase inhibitors (TKIs) as therapeutic agents. TKIs competitively bind and inhibit the catalytic kinase domain preventing EGFR from initiating signal transduction. Targeting EGFR in lung cancer is particularly successful in patients with activation mutations in ErbB1, while other NSCLC patients either are partially responsive, have disease stabilization, or do not respond at all [16-21]. Approximately 15% of tumors in lung cancer patients exhibit EGFR activating mutations and have significant responses to TKIs targeting EGFR. Resistant to EGFR inhibitors occurs and is associated with activation of additional signaling pathways, or secondary mutations in the ErbB1 gene that make EGFR less susceptible to inhibitors [20,22-27]. Resistance and lack of responsiveness in the majority of metastatic lung cancer patients emphasize the importance of identifying additional targets for drug therapy. In some tumor cell lines, EGF receptors are activated by unknown mechanisms, hence we reasoned that cell lines could be used to define additional proteins to target. Our approach was to delineate mechanisms of constitutive phosphorylation of EGFR in lung adenocarcinoma cell lines. In preliminary studies constitutive phosphorylation of the EGFR at Y-845 and Y-992 in the Calu3 cell line was found independent of EGF stimulation. The objective of this study thus, was to determine the mechanisms leading to constitutive phosphorylation of EGFR. Once the mechanisms are defined, then inhibitors can be selected to counteract constitutive receptor activation.

## Materials and methods

### Cell lines

Lung adenocarcinoma lines A549, A427, H2122, H1299, H1975 and Calu3 were obtained from ATCC. A549, A427 and Calu3 were grown in DMEM high glucose medium (Invitrogen) plus 10% fetal bovine serum (Gemini) and supplements of Minimal Nonessential Mineral & Vitamins, HEPES buffer, L-glutamine (Invitrogen) as recommended plus 0.75 μg gentimycin/ml. H1975, H1299, H2122 were grown in RPMI 1640 high glucose medium plus 10% FBS and 0.75 μg gentimycin/ml. Adherent cells were grown to confluency in T-25 or T-75 tissue culture flasks, washed in PBS, then detached with Cell Dissociation Buffer (Invitrogen). For inhibitor studies, Calu3 cells were seeded at 500,000 cells/well while H1975 cells were seeded at 750,000 cells/well and allowed to adhere overnight to achieve 80-90% confluency before serum starvation for 6 hours to overnight. Cells were treated with various inhibitors or solvent vehicles in serum-free medium as indicated.

### Reagents

AG1478 Tyrphostin (AG) (EGFR tyrosine kinase inhibitor), SU11274 (SU) (c-Met tyrosine kinase inhibitor), Diphtheria toxin mutant CRM197 [blocks HB-EGF and amphiregulin (AR)], and myristoylated PKCßII peptide inhibitor I (Sigma); erlotinib (Erl) [EGFR tyrosine kinase inhibitor, (Tarceva™) Genentech/Roche & OSI]; U0126 (U0) (MEK, and downstream Erk1/2 kinase inhibitor), and human recombinant EGF, [Cell Signaling Technology, Inc (CST)]; PP2 (Src-tyrosine kinase inhibitor), GM6001 (pan-metalloproteinase inhibitor) and TAPI (ADAM17 inhibitor) (Calbiochem); and Enzastaurin (LY317615, ENZA), [serine threonine kinase inhibitor of PKCß, (Lilly Oncology)]. Erlotinib (Tarceva™) and LY317615 (Enzastaurin) were obtained through Materials Transfer Agreements with OSI and Roche/Genentech, and with Lilly Oncology, respectively.

### Calcein AM proliferation assay

Cells were seeded at 15,000 cells per well into 96-well flat bottom plates. After adherence and serum starvation overnight, drugs or siRNA were diluted in serum free medium, and added to wells in triplicate then incubated at 37°C, 5% CO_2_ for 4–6 hours before an equal volume of Opt-MEM medium with 10% FBS but without antibiotics was added, then cultured for the length of times indicated. Two hours before harvesting, 100 μl of 4 μM BD™ Calcein AM was added to washed cells. Plates were read at 485 nm and relative fluorescence units (RFU) recorded. RFU of ten replicate wells were averaged and analyzed for significance. Mann Whitney unit analysis test was applied to relative fluorescent units (RFU) data from 10 replicate wells and p values are reported.

### Antibodies

Anti-EGFR, anti-phospho-EGFR (Y-845), anti-phospho-EGFR (Y-992), anti-phospho-EGFR (Y-1068), anti-phospho-HER3/ErbB3 (Y-1289), anti-phospho-Akt (Ser-473), anti-Akt, anti-phospho-GSK-3ß (Ser-21/9), anti-phospho-Src (Y-416), anti-Fyn, anti-Lyn, anti-Yes, anti-Lck, anti-Hck, anti-phospho-Lyn (Y507), anti-β-Actin and anti-phospho-p44/42 MAP kinase (Thr-202/Y-204) antibodies (Cell Signaling Technology); anti-ErbB2/HER2, anti-ErbB-3/HER3-clone 2 F12, anti-phospho-PKCβII (Y-641), and anti-human EGFR neutralizing antibody (LA1, Upstate Biotechnology); anti-phospho-c-Met (Y-1230,1234,1235) antibody (Invitrogen); mouse anti-RACK1 antibody (BD Biosciences); and rabbit anti-RACK1 and anti-Cbp/PAG antibodies (Santa Cruz Biotechnology). Mouse anti-Lyn, clone 10A6.2, and Milliplex® assays were from Millipore. Horse radish peroxidase conjugated secondary antibodies were: goat anti-rabbit Ig and goat anti-mouse Ig antibodies (Southern Biotech), anti-rabbit light chain TrueBlot®antibodies and anti-rabbit light chain TrueBlot IP beads® (E-Bioscience).

### Cell lysates

Inhibitors or equal volumes of DMSO solvent vehicle were added to adherent, serum-starved cells in 6-well plates before preparation of cell lysates. Where indicated, cells were stimulated with 500 or 100 ng/ml of human EGF (CST) for five-ten minutes at 37°C before medium was removed, and chilled cell lysis buffer immediately added. Dissolving cells were sonicated 15 seconds before microcentrifugation for 20 minutes. Supernatants were removed and protein concentrations quantitated using Bio-Rad Bradford protein assay. Generally 20–30 μg of protein were loaded into 7.5% Tris–HCl pre-cast SDS-PAGE gels (BioRad).

### MILLIPLEX® MAP 8-Plex phospho-Src (Tyr419) family kinase immunoassay

Quantitative sandwich immunobead assays (Millipore) were used to identify Y-419 phosphorylated SFK members including Src, Yes, Fyn, Fgr, Lck, Hck, Blk and Lyn. Cell-free lysates of unstimulated NSCLC cell lines were incubated with specific antibody conjugated beads which select a SFK member, followed by addition of biotinylated pan-anti-phospho-Src (Y419) to quantify the level of Y-419 phosphorylation of that SFK member. Samples were read in a luminex 100 reader after addition of PE-conjugated StrepAvidin. All assays were performed and analyzed with respect to a standard curve of Hela or Ramos cell lysates according to manufacturer recommended protocols.

### Western blotting

SDS-PAGE were performed using pre-cast 7.5% Tris–HCl gel (Bio-Rad) and electrophoresed in Tris Glycine-SDS buffer at 100 volts for 99 minutes. Separated proteins at 20–30 μg/lane were transferred to PVDF membranes using a semi-dry transfer apparatus (BioRad). Blotted membranes were washed, blocked overnight on a rocker at 4°C, then incubated with 1:1000 primary antibody diluted in SignalBoost™ (Calbiochem), 5% BSA, or 5% milk in TBST. Secondary antibodies were added at 1:2000 for 2 hours at 25°C. ECL substrate (Amersham Biosciences) was added, then blots exposed to film before developing. Anti-actin was used to control for equal protein loading after other antibodies were analyzed as stripping anti-phospho-blots and probing with anti-EGFR for example was not a reliable method.

### Immunoprecipitation

Two-five hundred μg of cell lysate proteins were incubated with 4 μg of antibody overnight on a rotator at 4°C. Recombinant Protein A/G ultra-link resin (Pierce) or Trueblot® anti-light chain IP beads (eBioscience) were washed and added at 1:10 ratio of beads to lysate volumes, then mixed further for 2–3 hours at 4°C. Immunoprecipitation mixtures were microcentrifuged for thirty seconds, the beads washed, then pellets resuspended in 20–65 μl 2x sample loading buffer, boiled, cooled, and microcentrifuged before loading 10–15 μl into SDS-PAGE gels.

### si-RNA transfection

Lyn siRNA (LYN ON-TARGET plus SMART pool) and negative control siRNA [ON-TARGET plus Non-Targeting siRNA pool, (Dharmacon)] were diluted to 250nM in antibiotic free OPTI-MEM with Glutamax (Invitrogen) and mixed with an equal volume of transfection reagent (DharmaFECT 2 diluted 1:40 in OPTI-MEM) then incubated 20 minutes at room temperature with shaking before 1.0 ml of each mixture was added to cells adhered to duplicate wells of a 6-well plate. Another 1.0 ml of OPTI-MEM containing 10% FBS but no antibiotics was added after 4–6 hours at 37°C, then the plates were incubated for 48, 72, 96, and 144 hours as noted. The kinetics and effectiveness of Lyn siRNA knock-down was confirmed by Western blotting with anti-Lyn or anti-phospho-Lyn. The sequences of the four Lyn siRNAs in the SMARTpools were 1) UUACAUCUCUCCACGAAUC; 2) GAGAUCCAACGUCCAAUAA; 3) GUGAUGUUAUUAAGCACUA and 4) GCGACAUGAUUAAACAUUA. The protocol to determine the effect of Lyn siRNA knock-down on Calu3 cell viability was modified to ten replicate wells in 96-well plates of Calcein AM assay as described above.

## Results

### Constitutive phosphorylation of EGFR in NSCLC cell lines

**Figure 1:**
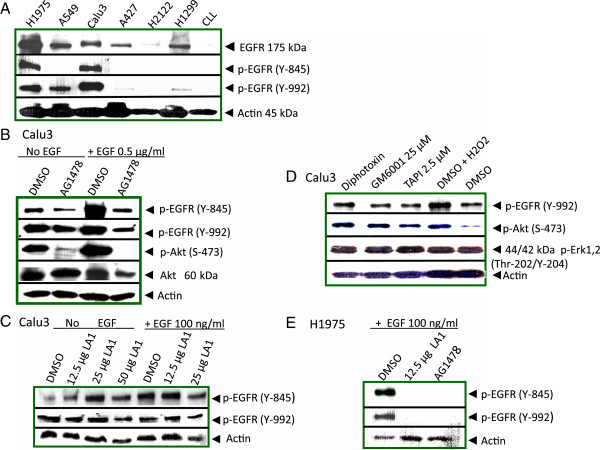
**Constitutive phosphorylation of EGFR in non-small cell lung cancer cell lines (NSCLC). (A)** Constitutive phosphorylation of EGFR at Y-845 and Y-992. Western blots from lysates of unstimulated NSCLC cell lines and chronic lymphocytic leukemia (CLL) cells were probed with anti-phospho-EGFR (Y-845), anti-phospho-EGFR (Y-992), anti-EGFR or anti-actin antibodies as loading controls. **(B)** EGFR triggered autophosphorylation is not responsible for constitutive EGFR (Y845) or (Y-992) phosphorylation in Calu3. Calu3 cells were incubated with EGFR kinase inhibitor @ 1 μM AG1478, or an equal volume of DMSO solvent, for 1 hour with or without addition of 0.5 μg EGF in the final 10 minutes before lysates were prepared and Western blotted with anti-phospho-EGFR (Y845 and Y-992), anti-phospho-Akt (ser-473), anti-Akt, or anti-actin. **(C)** EGFR ligands are not responsible for constitutive phosphorylation in Calu3 cells. EGFR neutralizing antibodies, LA1 at 12.5, 25 or 50 μg were incubated for 18 hours with Calu3 cells with or without 100 ng EGF in the final 5 minutes before lysates were prepared and Western blotted. **(D)** Transactivation by membrane associated ligands was not responsible for constitutive phosphorylation of EGFR Y-992 or downstream phosphorylation of Akt or Erk1,2. Calu3 cells were serum cultured with Corynebacterium diphtheriae toxin @ 10 μg/ml, 25 μM GM6001, 2.5 μM TAPI, 100 μM H_2_O_2,_ or an equivalent volume of DMSO for 1 hour before lysates were prepared and Western blotted. **(E)** EGFR neutralizing antibodies blocked phosphorylation in H1975 NSCLC cell line. LAI at 12.5 μg was added to H1975 cells for 18 hours. DMSO or 1 μM AG1478 was added for 1 hour. Lysates were prepared for SDS-PAGE and Western blotting with anti-phospho-EGFR(Y-992) and anti-phospho-EGFR (Y-845) or anti-actin. Caco-2 cells (ATCC #HTB-37, human colorectal adenocarcinoma) served as positive controls for the TACE (ADAM 17) inhibitors, GM6001 and TAPI [35] (data not presented).

Constitutive phosphorylation of EGFR at Y-845 in Calu3 and H1975 cell lines, and at Y-992 was seen in Calu3, H1975, and A549 cell lines (Figure 1A). CLL cells did not express EGFR and nonspecific staining with anti-phospho-EGFR antibodies was not observed. PCR and SSCP assays did not detect activating mutations in Calu3 cells in exons 19 and 21 of the *erbB1* gene (data not presented and [28]), hence Calu3 served as the target of our investigations. H1975 cells on the other hand contain an activating mutation in exon 21 resulting in EGFR phosphorylation.

To investigate mechanisms of constitutive activation of EGFR, autophosphorylation was inhibited with EGFR-tyrosine kinase inhibitor AG1478, and later confirmed with erlotinib. Phosphorylation of Y-992 and Y-845 of EGFR were still detectable in unstimulated, serum starved Calu3 cells confirming that they are not autophosphorylation sites, but are phosphorylated by upstream kinases (Figure 1B and data not presented) [29,30]. AG1478 was functional as it inhibited downstream phosphorylation of Akt (Ser-473). Ligands were not responsible for constitutive phosphorylation of EGFR in unstimulated, serum starved Calu3 cells as increments of EGF neutralizing monoclonal antibody, LA1, from 12.5 to 50 μg/ml failed to inhibit phosphorylation (Figure 1C). LA1, binds the EGFR extracellular domain and competes for binding with ligands; EGF, TGFα, and AR. LA1 was effective as it inhibited EGF-ligand induced Y-992 and Y-845 phosphorylation in H1975 cells (Figure 1E). Thus, phosphorylations regulated by activating mutations in H1975 cell line were susceptible to EGFR kinase inhibitors unlike constitutive phosphorylation in Calu cells.

Potential transactivation by autocrine triggered release of ligands including heparin binding-EGF (HB-EGF) and TNFα by metalloproteases was investigated [31-33]. ADAM17 is responsible for shedding of AR, TGFα, EPR, HB-EGF and HRG/NRG ligands from cell membranes [34]. TAPI, a TACE/ADAM17 specific inhibitor, and GM6001 a broad acting matrix metalloproteinase inhibitor, blocked the effects of metalloproteases on EGFR phosphorylation and signaling in Caco-2 control cells [35], but neither GM6001, nor TAPI, nor CRM-197, a diphthotoxin mutant which specifically prevents HB-EGF binding, blocked constitutive phosphorylation of Calu3 cells (Figure 1D). Constitutive activation of EGFR therefore was independent of transactivation via ADAM cleavage of membrane bound ligands and HB-EGF ligand stimulation. Taken together these results demonstrate that constitutive EGFR phosphorylations in Calu3 cells are independent of ligand binding and autophosphorylation. These results directed the study to focus on upstream intracellular kinases as the mechanism for constitutive phosphorylation of EGFR.

### Src family kinases (SFK) contribute to constitutive phosphorylation of EGFR

**Figure 2:**
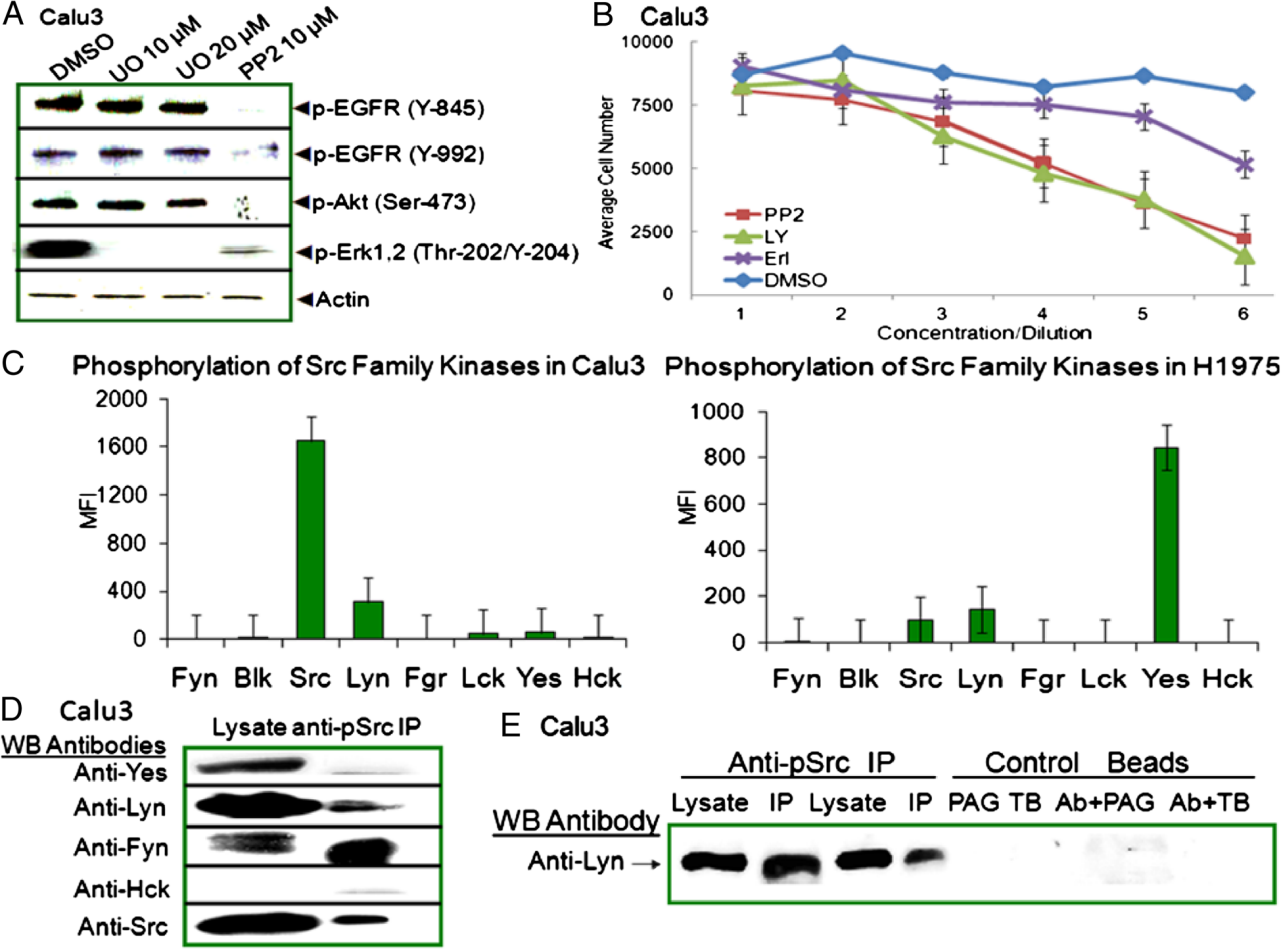
**Src Family Kinase (SFK) expression and activation. (A)** Src-kinase inhibitor PP2, blocked phosphorylation of EGFR at Y-845 and Y-992 and downstream phosphorylations of Akt and Erk1,2. Calu3 cells were serum starved for 6 hours, then cultured with 10 μM PP2, 10 or 20 μM U0126, or an equivalent volume of DMSO for 1 hour with addition of recombinant EGF @ 1 μg/ml for the final 10 minutes; then lysates prepared for SDS-PAGE and Western blotting with indicated antibodies. **(B)** PP2 decreased viability of Calu3 cells in a concentration dependent manner. Calu3 cells were cultured in triplicate in 96-well plates, and serum starved for 8 hours before addition of the respective inhibitors with highest concentrations for PP2 @ 20 μM; LY29004 @ 40 μM; Erlotinib @ 20 μM; and DMSO @ 4 μl; then dilutions of one-half through seven serial titrations (7 → 1). After 70 hours, Calcein AM was added two hours before harvesting. **(C)** Distinct patterns of SFK activation were revealed in a quantitative Milliplex assay of unstimulated Calu3 and H1975 cell lysates. MFI (median fluorescence intensity) equals sample - background. **(D)** Expression of phosphorylated Lyn, Src, and an isoform of Fyn in Calu3 cell lysates were confirmed by Western blotting. Anti-phospho-Src (Y-416) immunoprecipitates were blotted and probed separately with anti-SFK member antibodies including Yes, Lyn, Fyn, Hck, and v-Src. Anti-Hck served as a specificity control in that no non-specific bands were observed in either the lysates or IP. **(E)** Lyn expression and phosphorylation were further confirmed by direct immunoprecipitation. Calu3 cell lysates were incubated with anti-phospho-Src (Y-417), anti-vimentin isotype control or no antibody. Duplicate immunoprecipitations were performed with recombinant protein A\G conjugated beads (PAG) (wells 2,5,7) or TrueBlot ® anti-light chain beads (TB, wells 4,6,8). Control immunoprecipitations demonstrated no extraneous bands near mw of SFKs, 58–66 kDa.

SFK have been demonstrated in lung tumor tissues [36] and Src phosphorylates EGFR Y-845 in breast cancer cells [29,30]. The SFK inhibitor, PP2, ablated phosphorylation of EGFR at Y-845 and Y-992, eliminated downstream Akt phosphorylations, and decreased phosphorylated of Erk1,2 in Calu3 cells (Figure 2A). The decrease in EGFR phosphorylation was specific for SFK inhibition as the Mek/Erk1,2 inhibitor U0126 did not inhibit EGFR or Akt phosphorylation, but did block phosphorylation of Erk1,2 as reported. Calu3 cell viability was decreased by inhibition of SFKs in a PP2 concentration dependent manner (Figure 2B). Inhibition of downstream kinase, Akt, with LY29004 revealed a similar concentration-dependent decline in viability while substantially higher concentrations of the EGFR tyrosine kinase inhibitor, erlotinib, were required for an effect on viability. DMSO served as the solvent vehicle control.

Lyn and Src were identified as the major phosphorylated SFK members detected by the Milliplex® luminex assays in Calu3 cell lysates, while Yes was the major phosphorylated SFK member detected in H1975 (Figure 2C). The Milliplex system uses specific antibodies conjugated on beads to capture individual SFK members, followed by a biotinylated anti-phosphorylation specific antibody to quantitate phosphorylation of the captured Src family member (Figure 2C). Western blotting to identify individual SFK members used a reverse procedure where immunoprecipitations were performed with anti-phosphorylated Src (Y-416), then tested in Western blots with antibodies specific for individual Src family members. Lyn, Src and an isoform of Fyn were detected in immunoprecipitates from Calu3 lysates (Figure 2D). Yes was not phosphorylated while Hck was not detected. Control immunoprecipitations were performed with recombinant protein A/G beads, TrueBlot® anti-light chain beads, and isotype antibody controls to rule out nonspecific binding or heavy chain Ig contaminations. Extraneous bands were not observed in the molecular weight range of SFK members in the control immunoprecipitates, while Lyn was readily detected in anti-phospho-Src (Y-416) immunoprecipitates (Figure 2E).

### EGFR is physically associated with SFKs, c-Met, and other ErbB chains

**Figure 3:**
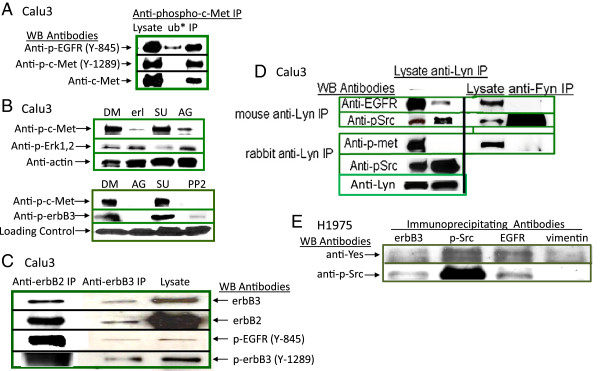
**EGFR is physically associated with distinct Src family kinases, c-Met, and other ErbB family members. (A)** Phosphorylated c-Met and EGFR from are pulled down together in anti-phospho-c-Met immunoprecipitations. Western blots of unstimulated Calu3 lysates, anti-phospho-c-Met (Y-1230,1234,1235) immunoprecipitates (IP), and “unbound” (*ub) proteins from IP supernatants were probed with anti-EGFR (Y-845) and anti-c-Met. While all phosphorylated c-Met was pulled down, a portion of phospho-EGFR remained, likely unbound to phosphorylated c-Met. **(B)** Unstimulated and serum-starved Calu3 cells were cultured with DMSO (DM) or 10 μM erlotinib (erl) for 3 hours, and 5 μM SU11274 (SU), 1 μM AG1478 (AG), or 10 μM PP2 for 1 hour before lysates and Western blots were prepared and probed with anti-phospho-c-Met (Y-1230,1234,1235), anti-phospho-Erk1,2 (Thr-202/Y-204), anti-phospho-ErbB3 (Y-1289), or anti-actin. **(C)** Immunoprecipitation of unstimulated Calu3 lysates with anti-ErbB3 also pulled down ErbB2, phosphorylated EGFR, and phosphorylated ErbB3. Anti-ErbB2 reciprocally immunoprecipitated ErbB3 from unstimulated Calu3 lysates. **(D)** Anti-Lyn pulled down EGFR but not phosphorylated c-Met (Left panels) while anti-Fyn pulled down neither EGFR nor c-Met (Right panels) from unstimulated Calu3 lysates. Western blots of mouse anti-Lyn IPs (top left panel) were probed with anti-EGFR and anti-phospho-Src (Y-416) while rabbit anti-Lyn IPs (lower left panels) were probed with anti-phospho-c-Met (Y-1230,1234,1235) and anti-phospho-Src (Y-416). **(E)** Yes was associated with EGFR but not ErbB3 in H1975. Lysates from untreated H1975 cells were immunoprecipitated with antibodies to ErbB3, phospho-Src (Y-416), EGFR, and vimentin. Western blots of the IPs were probed with anti-Yes and anti-phospho-Src (Y-416). Anti-vimentin immunoprecipitates served as specificity controls.

A physical association between phosphorylated EGFR and c-Met was confirmed in Western blots of anti-phospho-c-Met (Y-1230,1234,1235) immunoprecipitates where phosphorylated ErbB1 chains were pulled down with antibodies to phosphorylated c-Met (Figure 3A). EGFR kinase activity was responsible for c-Met phosphorylation as both erlotinib and AG1478, which target the tyrosine kinase domain of EGFR, inhibited phosphorylation of c-Met (Figure 3B). The inhibition of SFK activity with PP2 also inhibited phosphorylation of c-Met and of ErbB3 supporting an upstream activity for SFKs. The promiscuity of ErbB1 was further confirmed in anti-ErbB3 and anti-ErbB2 immunoprecipitates (Figure 3C). ErbB3 in the immunoprecipitates was activated by phosphorylation at Y1289. The physical association of ErbB1 with c-Met, ErbB2, or ErbB3 expands the network of signaling pathways that are activated in cancer cells and illustrates why a single tyrosine kinase inhibitor may not be sufficient to eradicate disease. An association with SFKs further enhances the spectrum of regulatory factors activated to alter gene expression in lung cancer cells and illustrates the importance of identifying the defining upstream triggering factor or kinase.

Since Lyn was highly expressed in the Calu3 lung cancer cell line, a role for Lyn in EGFR constitutive phosphorylation was investigated. Anti-Lyn antibodies pulled down EGFR demonstrating their physical association. Phosphorylated c-Met was not evident in anti-Lyn pull downs (Figure 3D). Different species of hosts for anti-Lyn production were used for immunoprecipitations to eliminate potential heavy chain contaminations identified by the secondary antibody in the Western blots, hence mouse anti-Lyn IPs were probed with rabbit anti-EGFR and pSrc while anti rabbit Lyn IPs were probed with mouse anti-p-c-met, Lyn and pSrc. While a phosphorylated Fyn isoform had been detected by immunoprecipitation, it had no physical association with either EGFR or c-Met (Figure 3D).

Western blots confirmed the presence of phosphorylated Yes in anti-phospho-Src (Y-416) immunoprecipitates of H1975 cell lysates (Figure 3E). Pull-down experiments revealed that EGFR was physically associated with Yes in H1975 cells as Yes was co-immunoprecipitated with anti-EGFR antibodies (Figure 3E). Anti-Vimentin IP served as a specificity control for the co-immunoprecipitations and no Yes or phosphorylated Src were non-specifically pulled down.

### Lyn contributes to NSCLC viability and signal transduction

**Figure 4:**
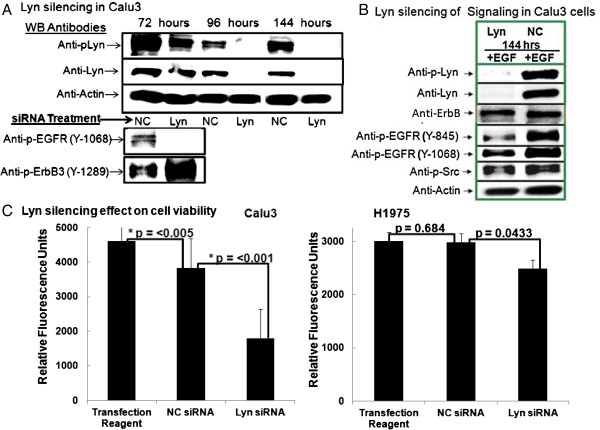
**Lyn siRNA inhibits EGFR activation, downstream phosphorylation of c-Met but not phosphorylation of ErbB3. (A)** Lyn specific siRNA inhibits Lyn and EGFR phosphorylation. Calu3 cells were cultured with 12.5 nM Lyn siRNA and NC siRNA for 72, 96, and 144 hours before lysates were prepared and 30 μg protein from each treatment loaded into SDS-PAGE wells for separation and Western blotting. Blots were probed with anti-phospho-Lyn, anti-Lyn, anti-phospho-EGFR (Y-1068), anti-phospho-ErbB3 (Y-1289), and actin. **(B)** EGF triggered reexpression and phosphorylation of Lyn was not evident after 144 hours of culture with 12.5 nM Lyn siRNA while NC siRNA had little effect on Lyn or EGFR phosphorylation levels. EGFR phosphorylations were substantially decreased by Lyn siRNA. Serum-starved Calu3 cells were treated with Lyn and NC siRNA, then cultured for 144 hours. EGF at 100 ng/ml was added for the final 10 minutes before lysates were prepared and 30 μg protein from each treatment loaded into SDS-PAGE wells for separation and Western blotting. Multiple blots were probed with antibodies against phospho-Lyn, non-phosphorylated Lyn control, phospho-EGFR (Y-845), phospho-EGFR (Y-1068), non-phosphorylated ErbB control, phospho-Src (Y-416) or β-actin. **(C)** Lyn siRNA @ 12.5 nM significantly decreased cell survival after 72 hours of culture. Lyn siRNA decreased viability to highly significant levels, p = <0.001 and 0.0433. Some nonspecific decrease in viability with NC siRNA was also evident but did not reach a similar level of significance. Calcein AM uptake was used to measure cell viability during the last 2 hours of culture. Mann Whitney unit analysis test was applied to relative fluorescent units (RFU) data from 10 replicate wells.

The importance of Lyn to EGFR signaling and cell viability was investigated by treatment of Calu3 cells with pools of 4 Lyn specific silencing RNAs and negative control siRNA. Decreased Lyn phosphorylation and protein expression were demonstrated in Western blots of kinetic studies with Lyn-siRNA transfection (Figure 4A). Decreased Lyn expression and phosphorylation readily inhibited Y-1068 autophosphorylation of EGFR. No decrease in phosphorylation of ErbB3 was observed. EGF stimulation of Calu3 cells after complete Lyn silencing at 144 hours demonstrated no ligand triggered phosphorylation of Lyn, and decreased phosphorylation of EGFR at the SFK-dependent Y845 phosphorylated site, as well as at Y1068 autophosphorylation site (Figure 4B). Lyn, Src, and EGFR phosphorylations remained evident in Calu3 cells transfected with negative control siRNA (Figures 4A and B).

A role for Lyn in cell survival was confirmed in that transfection with Lyn-siRNA significantly decreased unstimulated Calu3 and H1975 cell viability significantly in comparison to nonspecific inhibition of viability with nonspecific control (NC) siRNA (Figure 4C). Thus, Lyn plays a role in maintaining cell viability and signaling.

### Activation of Lyn and SFKs

**Figure 5:**
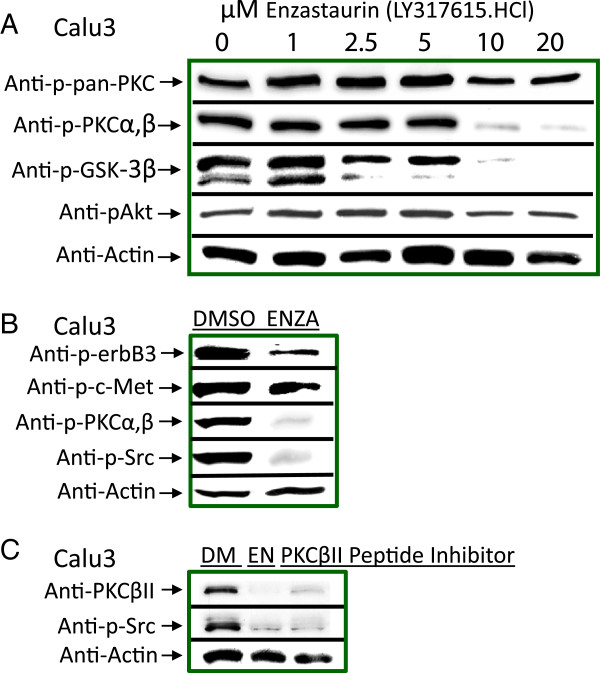
**PKCß inhibition reduced constitutive activation of Src, decreased phosphorylation of c-Met and ErbB3, and had downstream effects on Akt and GSK-3ß in both Calu3 and H1975 cells. (A)** PCKß inhibitor enzastaurin demonstrates that PKCß is required for Src activation. Concentrations of enzastaurin from 0–20 μM were added to unstimulated, serum-starved cells for 3.5 hours before lysates were prepared for Western blotting. Multiple blots were probed with antibodies against phospho-pan PKC, phospho-PKCα,ß, phospho-GSK-3ß, Akt and actin. **(B)** PKC inhibitor blocks constitutive activation. Unstimulated, serum-starved Calu3 and H1975 cells were cultured for 3.5 hours with 10 μM enzastaurin before lysates were prepared for Western blotting. Multiple blots were probed with antibodies against phospho-ErbB3 (Y-1289), phospho-c-Met (Y-1230,1234,1235), phospho-PKCα,ß, phospho-Src and actin. **(C)** Specificity of inhibition for PKCßII was confirmed by culture of unstimulated Calu3 cells with 80 μM PKCßII specific peptide inhibitor. Western blots were probed with anti-PKCβII antibodies, anti-phospho-Src (Y-416) and anti-actin.

Inhibition of EGFR phosphorylation by silencing Lyn RNA and a Src kinase specific inhibitor indicated that Src functions upstream to activate EGFR. The possibility that PKC was responsible for phosphorylating Src was investigated with enzastaurin, a serine-threonine kinase inhibitor that preferentially targets PKCβ. Concentrations of enzastaurin that inhibited PKCα,β phosphorylation led to decreased phosphorylations of EGFR downstream pathways including Akt and GSK-3β (Figure 5A). PKCα,β inhibition resulted in total inhibition of Src phosphorylation (Figure 5B). Since enzastaurin has secondary kinase targets, a more specific, cell-permeable, PKCβII peptide inhibitor was used and confirmed that PKCβII was responsible for regulating Src activation (Figure 5C). A PKCβII-dependent pathway therefore is responsible for SFK activation in Calu3 cells. Either PKCβII directly phosphorylates ser12 of Src, or indirectly results from its activation of CDK1/cdc2, or alternatively inactivates phosphatases that regulate SFK activity [37]. Peptide inhibitors function by binding their targets causing them to unfold, and subsequently become ubiquitinated, and proteosomally digested. The fact that little PKCβII protein was detected therefore demonstrates the effective inhibitory nature of the PKCβII peptide inhibitor (Figure 4C).

### Regulation of EGFR activation occurs in complexes with proteins associated with cell membranes

**Figure 6:**
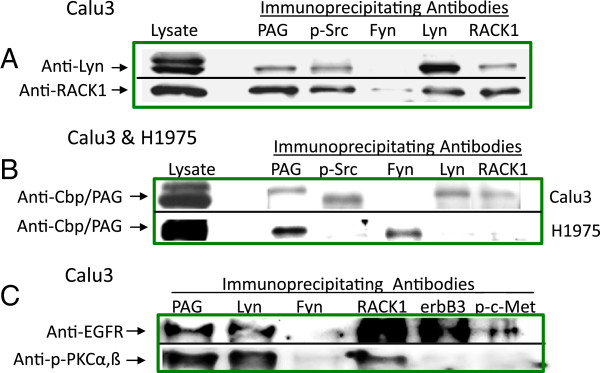
**Immunoprecipitations identify scaffolding and regulatory proteins pulled down with Lyn, PKCß, and EGFR. (A)** Anti-phospho-Src (Y-416), Lyn, RACK1 and Cbp/PAG co-immunoprecipitate each other. Immunoprecipitates from unstimulated, serum starved Calu3 cell lysates were performed with antibodies to Cbp/PAG, phospho-Src (Y-416), Lyn, Fyn and RACK1. Western blots were probed with anti-Lyn, and anti-RACK. No Lyn and minimally detectable quantities of RACK1 were immunoprecipitated by anti-Fyn. **(B)** Cbp/PAG was associated with phosphorylated Src, Lyn and RACK1 in Calu3 while anti-Fyn pulled down Cbp\PAG only in H1975 cells. Western blots of immunoprecipitates from unstimulated, serum starved Calu3 and H1975 cell lysates were probed with anti-Cbp/PAG. **(C)** EGFR, PKCα or β, Cbp/PAG, and RACK1 are all pulled down with anti-Lyn. Immunoprecipitates with antibodies to Cbp/PAG, Lyn, Fyn, RACK1, ErbB3 and phosphorylated-c-Met from Calu3 lysates were probed in Western blots with antibodies to EGFR and phospho-PKCα,ß.

Membrane scaffolding and Src-regulatory proteins, RACK1 and Cbp/PAG respectively, were investigated to determine whether they were in complexes with EGFR, PKCßII and Lyn. Both RACK1 and Cbp/PAG were detected in four NSCLC lines tested (data not presented) thus, immunoprecipitation experiments were undertaken to determine whether Lyn was associated with EGFR in complexes with Cbp\PAG and/or RACK1. A physical association between Lyn, RACK1, and Cbp/PAG in Calu3 cells was demonstrated in Western blotting of immunoprecipitates (Figure 6). Anti-Lyn co-immunoprecipitated RACK1 and Cbp/PAG. In reciprocal studies, both anti-Cbp/PAG and anti-RACK1 co-immunoprecipitated each other as well as Lyn (Figure 6A). Anti-Fyn antibodies did not co-immunoprecipitate Cbp/PAG or RACK1 from Calu3 cell lysates but did co-immunoprecipitate Cbp/PAG from lysates of H1975 cells (Figure 6B).

EGFR, a plasma membrane receptor, is physically associated with Lyn in Calu3 cells (Figure 3D). Lyn also associates with RACK1 and Cbp\PAG (Figure 6). Furthermore, PKCβII is required for phosphorylations of SFKs that include Lyn (Figure 4C). Thus, a series of pull-down experiments were performed to determine whether PKC, RACK1 and Cbp\PAG exist together with EGFR. Cbp\PAG partitions preferentially into membranes where it also associates with RACK1 which binds activated PKC. PKCα,ß was localized with Cbp\PAG, RACK1 and Lyn but not with Fyn, ErbB3 or phosphorylated c-Met (Figure 6C). Indeed, anti-Lyn pulled down both phosphorylated PKCα,β and EGFR (Figure 6C). PKCα,β was not detected in complexes reciprocally pulled down by either anti-p-c-Met or ErbB3. These studies thus suggest that EGFR associates with Lyn in membrane complexes of Cbp\PAG and RACK1 where PKCßII can affect Lyn or Src regulatory kinases and phosphatases resulting in activation of Lyn to phosphorylate EGFR and enhance its signaling activity.

## Discussion

The EGFR signal transduction pathway plays an important role in sustaining growth of lung cancer cells, yet therapy with TKIs is effective only in a subset of patients, thus we used lung adenocarcinoma cell lines to investigate mechanisms for constitutive phosphorylation of EGFR in order to identify additional targets for therapy. EGFR constitutive signaling in Calu3 cells was demonstrated to be ligand-independent. ADAM17 protein, an ErbB ligand sheddase, is upregulated and is required for EGFR and ErbB3 ligand-dependent signaling in NSCLC cell lines [38]. Yet, neither GM6001, a broad-range metalloprotease inhibitor, nor TAPI, a potent ADAM17 inhibitor, decreased EGFR phosphorylation at constitutive sites or downstream signaling confirming that cleavage of membrane associated ligands was not responsible for EGFR constitutive phosphorylation. Also, neutralizing antibodies did not block constitutive EGFR activation. Constitutive phosphorylation of EGFR thus was not due to ligand binding or transactivation.

Reportedly, SFKs phosphorylations of EGFR result in enhanced signaling potential [29,30,39,40], and SFKs were found to be responsible for EGFR constitutive activation (Figure 2). Lyn was physically associated with EGFR and identified as the specific SFK responsible for activating EGFR. While Lyn is preferentially expressed in normal and malignant B-cells, Lyn is also found in epithelial cells lining lung alveoli, and lining ducts from mammary, prostate and gut tissues [41-45]. Lyn was recently demonstrated as a requirement for internalization of microbial aggregates in lung epithelial cells and for responses to pathogens [46-48]. Mice deficient in Lyn expression, or transfected to overexpress Lyn, exhibit hyperactive B-cell receptor triggering, autoimmune diseases, and asthma-like symptoms in their lungs thereby emphasizing the importance of Lyn to lung physiology [49-51]. While the role for Lyn in leukemias and lymphomas is well established, a role for Lyn in solid tumors was only recently elaborated. Lyn was found to mediate tumor progression in head and neck squamous cell carcinomas, thyroid cancer growth and metastasis, sarcoma growth and survival, and a prognostic factor in colorectal cancer [52-55]. Lyn may serve therefore as a potential target for therapy in solid tumors.

Phosphorylated EGFR/ErbB1 chains are promiscuous as their physical associations with ErbB3, ErbB2, and c-Met were demonstrated in pull-down experiments (Figures 3 and 6C). These associations have functional consequences as inhibitor studies demonstrated that EGFR is responsible for phosphorylations of c-Met. Heterodimers also complicate EGFR targeted therapy as inhibition of EGFR enhances ErbB2/ErbB3 or EGFR/c-Met formation and activation [23,38,40,56]. SFKs also facilitate EGFR and c-Met heterodimer formation, and our studies emphasize the importance of SFKs to EGFR activation [Figures 2, 3, 4, 5 and 6] [36,41,42,57,58].

PKCßII was found to be critical to the downstream activation of EGFR, as PKCßII regulates activation of SFKs (Figure 5). PKCßII is known to regulate Src activation via CDK1/cdc2 and phosphatases [37]. Once activated, PKC becomes bound to the intracellular receptors, RACK1, stabilizing them within membrane lipid rafts where RACK1s then bind enzymes, substrates, growth factor receptors, integrins, and kinases [59-61]. RACK1 has been described as an inhibitory scaffold regulator of Src [62,63]. Activated SFKs and Src-regulatory kinases normally bind to Cbp/PAG which associates with glycosphingolipid-enriched microdomains (PAG) in membranes via palmitoylated tails [64-66]. Lyn can also become anchored in membrane lipids via myristoylation and palmitoylation, but in B-lymphomas Lyn has been localized to lipid rafts with Cbp/PAG [51,67-69]. In our studies, Cbp\PAG and Lyn were reciprocally co-immunoprecipitated demonstrating their physical association. A physical association between Lyn and EGFR, PKCα,ß, Cbp/PAG, and RACK1 was demonstrated in pull down experiments indicating that multiple signaling molecules form complexes or signalosomes with EGFR. RACK1 molecules can form homodimers with non-identical proteins bound to each so that one RACK1 partner could carry growth factor receptors such as EGFR, for example, while another could carry Lyn [70]. Alternatively Lyn could be brought into multi-protein complexes bound to Cbp\PAG as RACK1 and Cbp\PAG, Lyn and Cbp\PAG, were all reciprocally co-immunoprecipitated from Calu3 lysates (Figure 6). These data contrast with the EGFR mutationally activated H1975 cells where there was no evidence for co-immunoprecipitation of RACK1 and Cbp\PAG.

**Figure 7:**
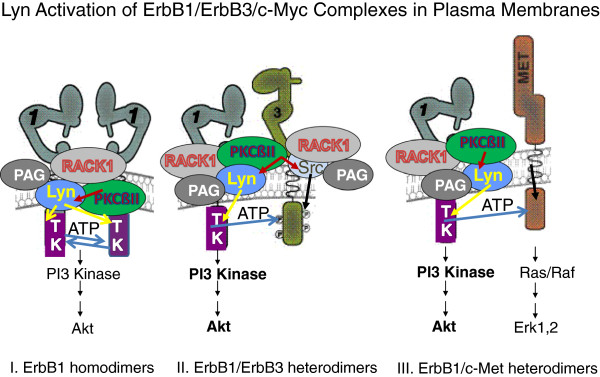
**Hypothetical working model of ErbB1 homodimers and heterodimers in lipid rafts with RACK1, PKCßII, Lyn and/or Src, Cbp/PAG. **EGFR/ErbB1 homodimers are drawn together, independent of ligand, within a single RACK1 complex while ligand-independent heterodimers of ErbB1 and ErbB3 or ErbB1 and c-Met may result from the merging of two or more RACK1complexes. Heterodimers lead to enhanced PI3 kinase activity as illustrated by bolding.

The interplay between RACK1 and Cbp\PAG is critical to Src family kinase regulation and to constitutive EGFR activation. Others have demonstrated that RACK1 binds the p110 active component of PI3Kinase, hence could bring PI3Kinase together with EGFR growth factor receptors to trigger downstream signaling [71]. In B-lymphoma lines, the p85 adaptor component of PI3Kinase was shown to bind to activated Cbp\PAG [68]. An association between Cbp\PAG and RACK1 thus could bring the two PI3Kinase components together such that activation of EGFR would trigger the PI3K cascade of signaling events. These latter studies emphasize the importance of scaffolding and\or adaptor proteins that pull receptors and kinases together within membrane complexes so that signals can be transduced. As a scaffolding protein, RACK1 would allow for the kinases to function in a multi-protein complex, and initiate a progression of activity to occur from PKCßII to activate Lyn, Lyn subsequently activating EGFR, followed by activation of PI3 kinase and c-Met, thus resulting in a cascading of signaling events (Figure 7). RACK1’s relevance to cancer progression was first demonstrated in breast cancer where its expression serves as an independent prognostic factor for poor outcome [71]. Elevated levels of Rack1 expression have been detected in lung cancer [72], and silencing of RACK1 expression has led to suppressed cancer cell growth and invasion both in vitro and in vivo [71,73]. In lung tumor cells that have ligand-independent, constitutively activated EGFR, targeting of scaffolding proteins such as RACK1 associated signaling complexes could result in the disruption of their functional capacities. Combining a Src kinase inhibitor with a drug targeting the scaffolding or adaptor proteins along with an EGFR TKI could break up the signaling unit thereby prevent further cell growth. Disruption of EGFR signalosomes could interfere with signaling even when ErbB1 is in promiscuous combinations with other ErbB family members, c-Met, or other receptor chains such as IGFR-1 [74-77]. Combination therapies to include disruption of signaling complexes thus could be a successful approach to eradicate lung cancer cells.

## Competing interests

The authors declare that they have no competing interests.

## Authors’ contributions

PS drafted portions of the manuscript and completed the laboratory research for, and constructed, the following figures: Figure 1A-E, Figure 2A, and Figure 3 A-C. Portions of this manuscript served as PS’s thesis for completion of a Master’s Degree in Medical Laboratory Science. JAB’s laboratory carried out the MILLIPLEX® MAP 8-Plex phospho-Src (Tyr419) family kinase immunoassay contributing the data presented in Figure 2C to define phosphorylated SKF members activated in the Calu3 and H1975 NSCLC cell lines. JAB and JMDP drafted that portion of the manuscript. PB contributed to the concept development by posing the initial question regarding the observation that approximately 50% of lung cancer patients whose tumors do not have activating EGFR mutations respond to TKI therapy directed against EGFR while another 50% do not; and provided financial support for the initiation of this project. JMDP’s research laboratory generated the vast majority of the data presented in this manuscript. JMDP guided the research by planning the experiments and analyzing the data. JMDP modified the initial draft of the manuscript with additional data, figures, text, analyses, and conclusions thereby completing the manuscript. All authors read and approved the final manuscript.
